# Editorial: Advanced therapeutic strategies and safety profiles in heart failure with reduced ejection fraction: contextualizing recent findings

**DOI:** 10.3389/fphar.2025.1607362

**Published:** 2025-04-28

**Authors:** Maria del Carmen Asensio-Lopez, Paolo Severino, Antonio Lax

**Affiliations:** ^1^ Cardiology Clinical & Experimental Group, Department of Medicine, Biomedical Research Institute (IMIB‐Pascual Parrilla), University of Murcia, Murcia, Spain; ^2^ Department of Research (CSO), Biocardio SL, El Palmar, Murcia, Spain; ^3^ Hemovascular Pathophysiology Research Group, Centro Nacional de Investigaciones Cardiovasculares (CNIC), Madrid, Spain; ^4^ Department of Cardiovascular, Respiratory, Nephrology, Anesthesiology and Geriatric Sciences, Sapienza University of Rome, Rome, Italy

**Keywords:** heart failure, therapy, reduced ejection fraction, medicine, pharmacology

## Introduction

Heart failure with reduced ejection fraction (HFrEF) remains a significant global health concern, characterized by high rates of morbidity, mortality, and hospitalization. Despite substantial progress in medical therapies over the past decades, the clinical course of HFrEF is often progressive, necessitating continuous research into novel and refined therapeutic strategies to improve patient outcomes. The management of HFrEF is a dynamic field, with clinical practice guidelines undergoing regular updates to incorporate the latest evidence and emerging treatment approaches. This constant evolution underscores the importance of ongoing research and the need for healthcare professionals to remain informed about the newest advancements in this area. The Research Topic “Advanced Therapeutic Strategies and Safety Profiles in Heart Failure with Reduced Ejection Fraction,” published in Frontiers in Pharmacology, serves as a valuable platform for the dissemination of cutting-edge research focused on enhancing the treatment and safety profile of individuals living with HFrEF. This Research Topic encompasses a wide range of investigations, including the application of artificial intelligence and machine learning for predicting therapeutic outcomes, detailed evaluations of drug efficacy and safety across diverse patient populations, comparative studies of different treatment strategies, clinical trials exploring novel pharmacological agents, and the integration of advanced diagnostic tools to personalize treatment responses. This editorial aims to critically analyze and contextualize three specific studies recently published in this Research Topic, integrating their findings into the current understanding of HFrEF management up to the year 2025. By examining these recent contributions, this editorial seeks to provide an expert perspective on their significance and potential impact on the evolving landscape of HFrEF therapy.

## Current landscape of HFrEF management

The cornerstone of contemporary HFrEF management lies in the implementation of guideline-directed medical therapy (GDMT), which has demonstrably improved survival and reduced hospitalizations (Heidenreich et al., 2022; Carrizales-Sepúlveda et al., 2024; Beghini et al., 2025). This fundamental approach is built upon the synergistic effects of four main classes of medications: Angiotensin Receptor-Neprilysin Inhibitors (ARNIs), which are often preferred as first-line renin-angiotensin system inhibitors due to their superior efficacy over ACE inhibitors or angiotensin receptor blockers; beta-blockers, which play a crucial role in mitigating the detrimental effects of the sympathetic nervous system; mineralocorticoid receptor antagonists (MRAs), which help to counteract the effects of aldosterone; and Sodium-Glucose Cotransporter-2 Inhibitors (SGLT2is), which have emerged as a transformative therapy demonstrating significant benefits in reducing heart failure hospitalizations and cardiovascular mortality in a broad range of patients with and without diabetes. Current guidelines increasingly emphasize the importance of initiating and rapidly up-titrating these four cornerstone therapies to their maximally tolerated doses within the first few months of diagnosis to achieve optimal clinical outcomes. Major guideline updates, such as the 2022 AHA/ACC/HFSA Guideline for the Management of Heart Failure and the 2023 ESC Focused Update of the 2021 ESC Guidelines, have further refined the recommendations for the management of HFrEF (Heidenreich et al., 2022). Notably, these updates highlight the expanded role of SGLT2 inhibitors across the entire spectrum of left ventricular ejection fraction, representing a significant shift in the therapeutic paradigm (Carrizales-Sepúlveda et al., 2024). Beyond these therapies, other emerging strategies and considerations have gained prominence in the management of HFrEF up to 2025 (Beghini et al., 2025).

Vericiguat, an oral soluble guanylate cyclase stimulator, is recommended for consideration in patients with worsening HFrEF despite optimal GDMT, based on the findings of the VICTORIA trial (PW et al., 2020). Intravenous iron supplementation is increasingly recognized for its benefits in symptomatic patients with HFrEF and iron deficiency, improving symptoms and quality of life and potentially reducing heart failure hospitalizations (Beghini et al., 2025). Furthermore, the integration of palliative care into the management of advanced heart failure is gaining increasing recognition as a crucial component in improving patients’ quality of life and addressing their individual goals of care (Carrizales-Sepúlveda et al., 2024). The 2024 ACC Expert Consensus Decision Pathway for Treatment of Heart Failure With Reduced Ejection Fraction further reinforces the central role of the “four pillars” of GDMT, emphasizing the preferred use of ARNIs as the initial renin-angiotensin system inhibitor in eligible patients. In this updated pathway, ACE inhibitors and angiotensin receptor blockers are primarily considered in patients with contraindications, intolerance, or limited access to ARNIs(Maddox et al., 2024).

## Analysis of recent studies

Three recent studies published in *Frontiers in Pharmacology* offer valuable insights into advanced therapeutic strategies and safety profiles in HFrEF, with their principal objectives, methodologies, and outcomes summarized below (Table 1).

**TABLE 1:** Summary of key findings from the analyzed studies.

Study (first author, year)	Objective	Methodology	Key findings	Primary implications
Li et al.	To assess the safety and efficacy of vericiguat in post-acute heart failure patients	A retrospective, single-center study comparing vericiguat users to a propensity-matched control group	Vericiguat was found to be safe and feasible in post-acute HF, even in the presence of hypotension/renal dysfunction; a potential renoprotective effect was observed	Suggests broader potential for vericiguat in a broader HF population beyond chronic HFrEF.
Huang et al.	To identify the most important drugs and drug classes associated with heart failure and acute heart failure	A retrospective pharmacovigilance analysis of the FDA Adverse Event Reporting System (FAERS) database (2004–2023)	Identified specific drugs and classes (e.g., antineoplastics, immunosuppressants, diabetes drugs) with high association with HF/AHF; early-onset risk observed	Reinforces the importance of drug-induced heart failure and the need for vigilance in prescribing, especially in high-risk patients
Xu et al.	To investigate the role of ATG5 in doxorubicin-induced cardiac toxicity	An in vivo study in mice using rAAV9 for ATG5 gene knockdown and overexpression; assessment of cardiac function and autophagy markers	ATG5 knockdown alleviated DOX-induced cardiotoxicity by inhibiting GATA4 degradation; DOX upregulated autophagy genes but inhibited flux	Highlights the ATG5-GATA4 pathway as a potential therapeutic target for the prevention of chemotherapy-induced cardiomyopathy

Study 1: Vericiguat in Post-Acute Heart Failure (Li et al.). This retrospective, single-center study sought to evaluate the safety and efficacy of vericiguat in 100 patients hospitalized for acute heart failure (HF) who were subsequently followed up as outpatients for a median of 68 days. These patients, representing a diverse group with HFrEF, HFmrEF, and HFpEF, were compared to a propensity score-matched external control group of 75 patients who received standard HF therapy without vericiguat during the same period. The study design, which included patients with various heart failure subtypes, broadened the scope of its findings beyond just HFrEF, potentially offering insights into the drug’s utility across the heart failure spectrum. The key findings of this study indicate that vericiguat is feasible and safe to use in patients following hospitalization for acute HF, even in those with pre-existing hypotension and renal dysfunction. Notably, the researchers observed a potential renoprotective effect associated with vericiguat, as evidenced by a slower decline in the estimated glomerular filtration rate (eGFR) in the vericiguat group compared to the control group. While both groups showed significant improvements in left ventricular ejection fraction (LVEF) and N-terminal pro-B-type natriuretic peptide (NT-proBNP) levels after treatment, the attenuation of eGFR decline in the vericiguat group was a particularly noteworthy observation. This finding is significant because renal dysfunction is a common and often complicating comorbidity in heart failure patients, frequently limiting treatment options. This study contributes valuable real-world data on the use of vericiguat in a broader heart failure population, including those with HFmrEF and HFpEF, in the immediate post-acute decompensation period. The evidence regarding the role of vericiguat in this specific setting is less robust compared to its established use in chronic HFrEF, where the VICTORIA trial demonstrated benefits in reducing cardiovascular death and heart failure hospitalization in high-risk patients (PW et al., 2020). The observation of a potential renoprotective effect is particularly interesting given the high prevalence of renal dysfunction in heart failure patients, which can often complicate treatment strategies (Lam et al., 2021). This finding aligns with preclinical studies suggesting that soluble guanylate cyclase (sGC) stimulators such as vericiguat may possess nephroprotective properties. The study’s findings on the feasibility and safety of vericiguat in this context add to the growing body of evidence supporting its use. The inclusion of patients with HFmrEF and HFpEF suggests that the benefits of vericiguat may extend beyond the current guideline recommendations, which focus primarily on its use in patients with worsening HFrEF despite optimal GDMT. The observed trend towards renal protection could have significant implications for the management of patients with heart failure and comorbid kidney disease, potentially improving long-term outcomes by mitigating the risk of renal deterioration.

Study 2: Drug-Induced Heart Failure: A Pharmacovigilance Study (Huang et al.). The second study in the Research Topic, by Huang et al., focused on “Drug-induced heart failure: a real-world pharmacovigilance study using the FDA adverse event reporting system database”. The primary objective of this retrospective pharmacovigilance analysis was to identify the top drugs and drug classes associated with heart failure (HF) and acute heart failure (AHF) as reported in the FDA Adverse Event Reporting System (FAERS) database from 2004 to 2023. The researchers analyzed a vast dataset of over 17 million adverse drug event reports, using specific search terms to identify those related to cardiac failure (Huang et al.). Their analysis revealed 38 specific drugs and 13 drug classes with a potentially high risk of causing HF, and 41 drugs and 19 drug classes associated with AHF. Notably, the top drug classes associated with HF included antineoplastic agents, immunosuppressants, antithrombotic agents, diabetes drugs, and antihypertensives, with diabetes drugs exhibiting the strongest association. For AHF, the leading drug classes were antineoplastic agents, antithrombotic agents, immunosuppressants, and drugs used for diabetes. The study also found that the median onset times for HF and AHF were 83 and 49 days, respectively, suggesting an early failure-type profile where the risk is highest in the initial stages of treatment with the implicated drugs. This relatively short time frame underscores the importance of early monitoring for cardiac dysfunction in patients starting these medications. This study emphasizes the recognized, but often underestimated, role of drug-induced heart failure. It reinforces the importance of post-marketing surveillance systems such as FAERS in identifying potential cardiotoxic effects of medications that may not be fully elucidated during pre-marketing clinical trials. The identification of specific drug classes and individual drugs with a high association with HF and AHF has significant implications for clinical practice, particularly when prescribing medications to patients with pre-existing cardiovascular risk factors for heart failure, warranting increased vigilance and monitoring (Maxwell and Jenkins, 2011). The findings align with existing knowledge regarding the cardiotoxicity of certain classes of drug, such as anthracyclines and some antiarrhythmic agents (Huang et al.). The strong association of diabetes drugs with heart failure, despite the known benefits of SGLT2 inhibitors in this population, suggests the need for further investigation of the cardiovascular safety profiles of other glucose-lowering agents. The early median onset times for HF and AHF highlight a critical period for monitoring patients initiating these medications, particularly those with pre-existing cardiac vulnerabilities. This information can inform clinical practice by prompting clinicians to have a heightened awareness of potential cardiac adverse events during the initial months of treatment with these specific drug classes. The overlap in drug classes associated with both HF and AHF may indicate common mechanisms of drug-induced cardiotoxicity, warranting further research to understand these pathways and develop preventive strategies.

Study 3: Knockdown of ATG5 Gene for Preventing Doxorubicin Cardiotoxicity (Xu et al.). The third study in this Research Topic investigated the role of the ATG5 gene in doxorubicin (DOX)-induced cardiotoxicity. Doxorubicin is a widely used chemotherapeutic drug, but its cardiotoxic mechanisms are not fully understood. Previous studies have indicated that autophagy activation is essential in DOX-induced cardiac toxicity, but the specific role of autophagy protein 5 (ATG5) has remained limited. Therefore, this study aimed to elucidate the role of ATG5 in this process. To establish a cardiac toxicity model, mice were intravenously administered DOX (5 mg/kg) for 4 weeks. The researchers then assessed cardiac function using echocardiography and analyzed cardiac tissue for protein expression, mRNA levels, fibrosis, and immunofluorescence staining. They also utilized recombinant adeno-associated virus serotype 9 (rAAV9) vectors to achieve both knockdown (shRNA-ATG5) and overexpression (ATG5) of the ATG5 gene in the mice. Additionally, Bafilomycin A1 was used to assess autophagic flux. The main findings of the study revealed that DOX treatment upregulated the expression of autophagy-related genes but paradoxically inhibited autophagic flux both in vitro and in vivo. The DOX-treated mice exhibited decreased cardiac function and cardiomyocyte size, along with increased cardiac fibrosis, oxidative stress, and apoptosis. Importantly, these detrimental effects of DOX were partially alleviated by the knockdown of the ATG5 gene using rAAV9-shRNA-ATG5 and were exacerbated by the overexpression of ATG5 using rAAV9-ATG5. The study further demonstrated that ATG5-mediated autophagy promoted the degradation of the GATA4 protein, a transcription factor known to protect against DOX-induced cardiotoxicity. Knocking down ATG5 or inhibiting autophagy chemically led to increased GATA4 protein expression, while overexpressing ATG5 or activating autophagy resulted in decreased GATA4 levels. Moreover, enforced re-expression of GATA4 significantly counteracted the toxic effects of ATG5 on DOX-treated hearts. The study concluded that manipulating ATG5 expression to regulate GATA4 degradation in the heart may be a promising therapeutic approach for preventing or mitigating DOX-induced cardiac toxicity. This research has significant implications for the field of cardio-oncology, suggesting a potential avenue for reducing the incidence of heart failure in cancer survivors who have received doxorubicin. The finding that DOX disrupts autophagic flux, despite upregulating autophagy-related genes, highlights a complex mechanism of cardiotoxicity where the initial cellular response is not effectively completing the clearance of damaged components. The identification of ATG5 as a key regulator of GATA4 degradation provides a specific target for potential therapeutic interventions aimed at protecting the heart during doxorubicin chemotherapy. The successful use of rAAV9 for targeted gene delivery in the heart in this preclinical study suggests the potential for future translation of these findings into clinical therapies.

## Contextualizing the recent findings within HFrEF management

The findings from the three analyzed studies offer distinct yet complementary perspectives on the evolving landscape of HFrEF management. The study by Li et al. provides valuable real-world evidence supporting the potential use of vericiguat beyond its current established role in chronic, worsening HFrEF despite optimal GDMT. While current guidelines recommend vericiguat for such patients based on the VICTORIA trial, this recent study suggests that vericiguat may be safe and feasible in a broader post-acute heart failure population, including those with HFmrEF and HFpEF. The observed potential renoprotective effect is particularly noteworthy, as renal dysfunction is a common and significant comorbidity in heart failure. If confirmed by further research, this finding may influence future guideline updates and expand the clinical scenarios in which vericiguat may be considered beneficial. This could represent a shift towards considering vericiguat earlier in the management of heart failure patients, especially those at risk of or with existing kidney disease. The pharmacovigilance study by Huang et al. underscores the critical importance of considering drug-induced cardiotoxicity in the management of patients with or at risk of HFrEF. The identification of specific drug classes, particularly antineoplastic agents, immunosuppressants, and diabetes drugs, as having a strong association with heart failure and acute heart failure reinforces the need for careful review of medication history and risk assessment. This information has direct implications for prescribing practices, emphasizing the need for increased awareness and monitoring when using these medications, especially in individuals with pre-existing cardiac conditions or risk factors. The relatively short median onset times for drug-induced heart failure highlight the importance of early vigilance for cardiac symptoms after initiation of these medications. This study emphasizes the need for a collaborative approach among different medical specialties, such as cardiology, oncology, and endocrinology, to optimize medication regimens and minimize the risk of drug-induced cardiotoxicity. The research by Xu et al. on doxorubicin-induced cardiotoxicity offers significant insights into the prevention of heart failure in cancer survivors. By identifying the ATG5-GATA4 pathway as a key mechanism in this process, the study paves the way for the development of novel cardioprotective strategies. The preclinical findings suggest that modulating ATG5 expression to prevent GATA4 degradation may be a promising approach to mitigate chemotherapy-induced cardiomyopathy, a significant concern that can lead to HFrEF in cancer survivors. The potential for translating these findings into clinical trials of ATG5 modulators or GATA4-enhancing therapies in patients undergoing doxorubicin chemotherapy is substantial. Preventing chemotherapy-induced cardiomyopathy is crucial for improving the long-term cardiovascular health and quality of life of cancer survivors, and this research offers a potential avenue to achieve this goal. This study highlights the intricate molecular mechanisms underlying drug-induced cardiotoxicity and the potential for targeted therapies to prevent or reverse these effects.

## Conclusion

The three studies analyzed in this editorial add valuable knowledge to the understanding and management of HFrEF. The vericiguat study suggests a potential extension of its clinical utility in the post-acute heart failure setting and suggests a possible renoprotective effect. The pharmacovigilance study reinforces the importance of considering drug-induced cardiotoxicity and highlights specific classes of drugs that warrant careful monitoring. Finally, the research on doxorubicin-induced cardiotoxicity identifies a promising therapeutic target for the prevention of heart failure in cancer patients. Collectively, these studies underscore the ongoing advancements in HFrEF therapy, ranging from optimizing the use of existing medications in broader patient populations to identifying novel therapeutic targets to prevent cardiotoxicity. Future research, including larger-scale clinical trials, will be crucial to validate these findings and further explore their implications for improving outcomes in patients with, or at risk for, heart failure with reduced ejection fraction.

## References

[B1] Beghini A. Sammartino A. M. Papp Z. von Haehling S. Biegus J. Ponikowski P. (2025). 2024 update in heart failure. Esc. Hear. Fail. 12, 8–42. 10.1002/EHF2.14857 PMC11769673 38806171

[B2] Carrizales-Sepúlveda E. F. Ordaz-Farías A. Vargas-Mendoza J. A. Vera-Pineda R. Flores-Ramírez R. (2024). Initiation and up-titration of guideline-directed medical therapy for patients with heart failure: better, Faster, stronger. Card. Fail. Rev. 10, e03. 10.15420/CFR.2023.20 38533397 PMC10964286

[B3] Heidenreich P. A. Bozkurt B. Aguilar D. Allen L. A. Byun J. J. Colvin M. M. (2022). 2022 AHA/ACC/HFSA guideline for the management of heart failure: a report of the American College of cardiology/American heart association Joint Committee on clinical practice guidelines. Circulation 145, E895–E1032. 10.1161/CIR.0000000000001063 35363499

[B4] Lam C. S. P. Mulder H. Lopatin Y. Vazquez-Tanus J. B. Siu D. Ezekowitz J. (2021). Blood Pressure and safety events with vericiguat in the VICTORIA trial. J. Am. Heart Assoc. 10, e021094. 10.1161/JAHA.121.021094 34743540 PMC8751950

[B5] Maddox T. M. Januzzi J. L. Allen L. A. Breathett K. Brouse S. Butler J. (2024). 2024 ACC expert Consensus decision pathway for treatment of heart failure with reduced ejection fraction: a report of the American College of cardiology Solution Set Oversight Committee. J. Am. Coll. Cardiol. 83, 1444–1488. 10.1016/J.JACC.2023.12.024 38466244

[B6] Maxwell C. B. Jenkins A. T. (2011). Drug-induced heart failure. Am. J. Health. Syst. Pharm. 68, 1791–1804. 10.2146/AJHP100637 21930637

[B7] Pw A. B P. Kj A. J E. Af H. J B. (2020). Vericiguat in patients with heart failure and reduced ejection fraction. N. Engl. J. Med. 382, 1883–1893. 10.1056/NEJMOA1915928 32222134

